# Hepatocellular carcinoma patients serum modulates the regenerative capacities of adipose mesenchymal stromal cells

**DOI:** 10.1016/j.heliyon.2024.e24794

**Published:** 2024-02-01

**Authors:** Radwa Ayman Salah, Azza M. El-Derby, Zaynab El-Gammal, Bishoy Wadie, Sara M. Ahmed, Shimaa E. Elshenawy, Shireen Magdy, Ayman Salah, Mahmoud Gabr, Ihab Mohamed, Nagwa El-Badri

**Affiliations:** a Center of Excellence for Stem Cells and Regenerative Medicine, Zewail City of Science and Technology, Giza, 12578, Egypt; b Stem Cells and Regenerative Medicine Department, Egypt Center for Research and Regenerative Medicine (ECRRM), Giza, 12578, Egypt; c Department of Hepatogastroenterology, Kasr El-Aini Cairo University, Cairo, Egypt; d Urology and Nephrology Center, Mansoura, 35516, Egypt; e Department of Zoology, Faculty of Science, Ain Shams University, Cairo, 11566, Egypt

**Keywords:** Adipose mesenchymal stem cells, HCC serum, huh7 cells, Hepatic cancer cell line

## Abstract

Hepatocellular carcinoma (HCC) is one of the most prevalent cancers causing the highest mortality rate worldwide. Treatment options of surgery, radiation, cytotoxic drugs and liver transplantation suffer significant side effects and a high frequency of relapse. Stem cell therapy has been proposed as a new effective therapy, however, controversial reports are emerging on the role of mesenchymal stem cells in cancer.

In this work, we aimed to assess the regenerative capacities of adipose mesenchymal stem cells when exposed to serum from HCC patients, by assessing the effect of the sera on modulating the regenerative capacities of h-AMSCs and the cancer properties in HCC cells. This will pave the way for maximizing the efficacy of MSCs in cancer therapy.

Our data show that HCC serum-treated hA-MSCs suffered oncogene-induced senescence as shown by their altered morphology and ameliorated proliferation and differentiation. The cells were enlarged with small irregular nuclei, swollen rough endoplasmic reticulum cisternae, and aging lysosomes typified by dark residual bodies. HCC serum-treated Huh-7 cancer cells on the other hand displayed higher tumor aggressiveness as depicted by altered morphology, increased cellular proliferation and migration, and decreased percentage of early and late apoptotic cells.

Our findings provide evidence that exposure of hA-MSCs to the serum of HCC patients decreases their regenerative capacities and should be considered when employed as a potential therapy in HCC patients.

## 1 Introduction

### 1.1 Background

Hepatocellular carcinoma (HCC) is one of the most prevalent cancers worldwide with high patient morbidity and mortality [1]. Since only 10–20 % of HCC patients are eligible for surgical intervention at the time of diagnosis, liver transplantation remains their first treatment choice [2]. A high frequency of relapse is reported, and the postoperative 5-year survival is as low as 18.4 % in patients who had performed curative resection [3]. Recent therapeutic approaches such as stem cell therapy have been proposed to control the disease at various stages [4].

Mesenchymal stem cells (MSCs) are multipotent cells with high regenerative capacities and can differentiate into different cell types [[5], [6], [7]]. MSCs could be isolated from the adipose tissue, bone marrow, umbilical cord and other organs and tissues [8,9]. MSCs have been proposed for treating cancer due to their immune modulating properties and low immunogenicity. However, MSCs are also one of the first cells to be recruited to the tumor site to fight cancer. They were reported to promote apoptosis, suppress HCC growth, invasion and migration both *in-vitro* and *in-vivo* [4]. MSCs were also reported to have an *in-vivo* hepatic differentiation potential, supporting their therapeutic role in liver regeneration [10,11]. MSCs have also been shown to downregulate proinflammatory and fibrogenic cytokines, to stimulate hepatocellular proliferation, and to inhibit hepatocyte apoptosis and fibrosis by collagen degradation [12]. The immunosuppressive effects of MSCs works synegystically to diminish the immunosuppressive drugs administered to given to liver transplant patients, and this may play a role in decreasing their morbidity and mortality [4,11,[13], [14], [15]]. Bone marrow (BM)-MSCs were reported to promote apoptosis, suppress HCC cell proliferation, migration and inhibit tumor growth via activation of P21 and caspases in tumor cells [16,17]. Decreased tumor invasion and metastatic formation were also reported via down regulation of TGFB1 and MMP-2 [18]. It was suggested that exporting miR-122 via hA-MSC exosomes can significantly enhance HCC chemosensitivity and may provide a more effective treatment option [19]. Additionally, a better therapeutic effect for MSCs was reported in HCC rat models when administered with Melatonin, attributed to triggering apoptosis and targeting inflammation in HCC [20]. In another xenograft HCC model, a combination of the chemotherapeutic agent, Sorafenib, and MSC was shown to yield more favorable results in the treatment of HCC [21]. Despite the accumulating data on the anti-cancer properties of MSCS, their applicationsn in liver patients was limited to liver cirrhosis [22], and their use for liver cancer in clinical trials remains rare [4]. Currently, four clinical trials were performed involving stem cells in HCC (clinicaltrials.org).

In liver tissues, MSCs constitute important component of the tumor microenvironment (TME). MSCs are recruited first to attack the cancer cells, but as the disease progresses, they play an important role in cancer development and spread [[23], [24], [25], [26]]. MSCs promote tumor growth and metastasis via sustaining angiogenesis, maintaining the cancer stem cell (CSC) niche, and exerting immune protection [[26], [27], [28], [29]]. Activation of the Wnt/β-catenin pathway may be involved in HCC initiation, increased proliferation and invasiveness [16,30]. MSCs were also found to activate the IL-6/STAT3 signaling pathway which results in promoting invasion and metastasis in HCC cell lines [31]. BM-MSCs were reported to promote metastasis and proliferation of human hepatoma cell line Bel7407 both *in-vitro* and *in-vivo* through upregulation of MAPK signaling pathway [32]. Another study reported that HCC-associated MSCs enhanced tumor spheroid formation and CSC marker expressions *in-vitro* [33]. HCC soluble factors were shown to induce a cancer-associated phenotype, genotype and functionality in MSCs [34,35].

HCC patient serum was found to be rich in growth factors, exosomes, extracellular vesicles, chemokines, and inflammatory factors that serve as diagnostic markers for HCC and were linked with the disease's biological makeup and its progression [[36], [37]]. Upon the delivery of the circulating genetic factors of cancer serum to non-malignant cells, they were shown to acquire different characteristics, including malignant traits [38]. Treatment of immortalized human embryonic kidney cell line (hEK293) with cancer sera resulted in a significant increase in metabolic activity, angiogenesis, tumor formation, cell proliferation, and transferred tumor traits when compared to treatment with healthy patient serum [38]. In contrast, normal cells (human MSCs and human adult liver fibroblasts (hALFs)) opposed the transformation caused by cancer patient sera. This suggests that transformation requires firstly primed cells, and that the therapeutic potential of MSCs varies significantly and depends on the TME [38].

In this study, we aimed to investigae the effect of sera from HCC patients on modulating the regenerative capacities of h-AMSCs to asses their fate and plasticity within the tumor stroma. We also investigated the effect of sera from HCC patients on maintaining the cancer properties in liver cancer cells to better understand the cross talk between cancer cells and the TME, and enhance the therapeutic efficacy of MSCs in cancer.

## 2 Material and methods

### 2.1 Sample collection and inclusion criteria

Blood samples were collected from 15 HCC patients and 15 healthy volunteers from the Faculty of Medicine Cairo University, and the National Liver Institute Monoufia University. HCC patients undergoing liver transplantation in Kasr El-Aini Hospital, Faculty of Medicine Cairo University, Egypt included males and females with an age >45 years. HCC was diagnosed via blood tests measuring liver functions, CT scan, and liver biopsy. Healthy donors included males and females with an age ≥30 years with no personal/family history of cancer and screened negative for hepatitis B and C viruses. The protocol was approved by the Institutional Review Board (IRB) of both institutes, and informed consent forms were taken from subjects who screened negative for blood-borne infections.

### 2.2 Cell culture and treatment

Huh-7 cells lines and hA-MSCs (a kind gift from the School of Medicine New Giza University (NGU), Giza, and the Urology and Nephrology Center, Mansoura University, Manoura, Egypt respectively), were maintained in Dulbecco's Modified Eagle Medium (DMEM) (Life Technologies, USA) supplemented with 5 ml (10 %) fetal bovine serum (FBS) (Life Technologies, USA), penicillin and streptomycin (Life Technologies, USA) at 37 °C and 5 % CO_2_. Serum samples were collected from HCC patients and healthy volunteers by allowing blood to clot at room temperature for 60 min, then centrifugation was performed at 3000×*g* for 10 min and stored at −80 °C until further use. Serum samples were pooled for each group respectively. 8 × 10^4^-1x10^5^ hA-MSCs and 5 × 10^4^-6 x10^4^ Huh-7 cells were cultured in each well of a 6-well culture plate (Greiner, Germany) in the presence of either HCC patient serum or normal human serum (control), which had been filtered through 0.2 μm filters (Millipore, USA), for 6 days and 21 days. Cells were maintained in Dulbecco's DMEM supplemented with 5 ml (10 %) human serum, penicillin and streptomycin at 37 °C and 5 % CO_2._ The experimental groups included normal serum-treated hA-MSCs, HCC serum-treated hA-MSCs, normal serum-treated Huh-7, and HCC serum-treated Huh-7 cells.

### 2.3 Immunostaining characterization of pluripotency markers

All groups were cultured on glass slides that were pre-coated with poly-d-lysine (PDL) (Sigma-Aldrich, USA). Using 4 % paraformaldehyde, 0.1 % TritonX-100, and 4 % BSA, cells were fixed, permeabilized, and blocked respectively. Cells were then stained with cell-specific antibodies anti-*OCT4* (R&D Systems, USA) at dilution 1:100 μl, anti-SOX2 (R&D systems, USA) at dilution 1:200 μl, and anti-NANOG (Bioss Antibodies, USA) at dilution 0.5:100 μl, and their appropriate secondary antibodies (Invitrogen, USA) at dilution 0.5:100 μl. To visualize the nucleus, cells were stained with Hoechst 33342 (Life Technologies, USA) at dilution 1:1000 μl. Cells were imaged using Leica inverted fluorescence microscopy (LEICA DMi8).

### 2.4 Ultrastructure characterization by electron microscopy

The cells were fixed by adding 0.1 M cacodylate-buffered 2 % glutaraldehyde at 4 °C then were washed with equal volumes of 0.4 % sucrose and 0.2 % cacodylate for 2 h. Cells were then fixed with equal volumes of 0.2 % cacodylate and 2 % osmic acid for 1 h, then washed with distilled water twice. Next, cells underwent dehydration by incubating them with an ascending series of ethyl alcohol for 5 min each, then clearing them with propylene oxide. Finally, cells were embedded in epoxy resin blocks. These blocks were cut into 1 μm sections using an ultramicrotome (American Optical Co., USA). These sections were examined under light microscopy after being stained with methylene blue and azure mix. These sections were further cut into 60 nm sections using the same ultramicrotome. Then, sections were placed on perforated copper grids (Electron Microscopy Science, Hatfield, USA) and stained with uranyl acetate and lead citrate. A transmission electron microscope (TEM, Philips EM 208S, Tokyo, Japan) was used to examine and image cells at an acceleration voltage of 80 kV.

### 2.5 Flow cytometry characterization

The phenotypic characterization of normal serum-treated hA-MSCs and HCC serum-treated hA-MSCs was performed using flow cytometry after 6 days. To prepare the cells, hA-MSCs were blocked with 1 % BSA for 10 min, centrifuged, and re-suspended in the blocking buffer. Cells were then stained with the following monoclonal antibodies for 30 min: PE anti-CD90, FITC anti-CD73, and APC anti-CD45. Flow cytometry was conducted using FACSCalibur™ (Becton DickiNSon) following standard procedures using CellQuest Pro Software (Becton DickiNSon). Data analysis was processed using FlowJo v. 10.2 software (Treestar, USA) with super-enhanced Dmax (SED) subtraction analysis for the detection of differences in histograms. All the experiments were conducted in triplicates.

### 2.6 Genotypic analysis using quantitative real-time polymerase chain reaction (qRT-PCR)

RNA of the cells was extracted by Trizol (Qiagen, Germany). cDNA was prepared using 10 μl of total RNA, 1 μl antisense primer (20 pmol), and 1 μl reverse transcriptase enzyme for 15 min at 42 °C. To quantify the expression of specific genes, 5 μl of the cDNA along with 2× SYBR Green PCR Master Mix (Applied Biosystems, USA) and 5 pmol of each primer (Table S1) were used. cDNA was amplified as follows: the initial denaturation step for 15 min at 95 °C, followed by 40 cycles of denaturation (15 s at 94 °C), annealing (60 °C for 30 s), and extension (30 s at 72 °C). The normalized expression of relevant genes was calculated using ΔCTmethod with β-actin gene as a housekeeping gene.

### 2.7 Side population assay

hA-MSCs and Huh-7 treated with normal and HCC patients’ sera were trypsinized, and re-suspended in DMEM supplemented with 2 % FBS. The nuclei of the cells were stained with 5 μg/ml of Hoechst 33342 dye (Molecular Probes, USA) for 90 min at 37 °C, centrifuged for 10 min at 1800 rpm, and the pellet was re-suspended in HBSS containing 2 % FBS. Just before the acquisition of the flow cytometry (Thermofisher, USA), two μg/ml propidium iodide (Sigma-Aldrich, USA) were added. The excitation wavelength of Hoechst dye was 350 nm (UV laser).

### 2.8 Cell cycle analysis

hA-MSCs and Huh-7 cells were trypinized and redissolved in ice-cold Phosphate Buffer Saline (PBS) without Ca2+, Mg2+ (PAN Biotech, Germany). These cells were then fixed overnight by chilled 70 % ethanol at 4 °C. Cells were then stained in PBS containing 50 μg/ml propidium iodide (Sigma Aldrich, USA) and 100 μg/ml RNase A (Thermofisher Scientific, USA). Flow cytometry was performed using a FACS Calibur (Becton Dickinson, USA) following standard operating procedures, and cells were analyzed using Cell Quest Pro Software (Becton Dickinson, USA).

### 2.9 Apoptosis analysis

Apoptosis assay was conducted for hA-MSCs and Huh-7 cells treated with normal or HCC sera for 6 days. Apoptosis was assessed using Annexin-V-FITC and propidium iodide (PI) apoptosis detection kit (Miltenyi Biotec Inc., USA) according to the manufacturer protocol. All experiments were performed in triplicates.

### 2.10 Differentiation assay

Adipogenic and osteogenic differentiation were examined for hA-MSCs cultured in normal and HCC sera as previously mentioned [30]. To induce the osteogenic differentiation, hA-MSCs were cultured in an osteogenic differentiation medium (DMEM, 10 % FBS, 2 mM l-glutamine, 100 U/ml penicillin-streptomycin amphotericin, 0.1 μM dexamethasone, 50 μM l-ascorbic acid, and 10 mM β-glycerophosphate) for 21 days (Lonza, USA; Serva, Germany; Sigma Aldrich, USA). To detect calcium deposits, Alizarin red staining (Alpha Chemika, India) was used. To induce the adipogenic differentiation, hA-MSCs were cultured in an adipogenic differentiation medium (DMEM, 10 % FBS, 2 mM l-glutamine, 100 U/ml penicillin-streptomycin amphotericin, 0.5 mM isobutyl-methyl-xanthine, 1 μM dexamethasone, 10 μM insulin, and 200 μM indomethacin (Lonza, Switzerland; Sigma, USA; Serva, Germany; Acros Organics, USA) for 14 days. To detect the lipid droplets, Oil red O staining (Acros Organics, USA) was used.

### 2.11 MTT assay

To assess the proliferation of the cells, cells were cultured in a 6-well plate, treated with 5 mg/ml MTT reagent 3-(4,5-dimethylthiazol-2-yl) −2,5-diphenyltetrazolium bromide (Life Technologies), and incubated for 3 h in a humidified 5 % CO_2_ incubator at 37 °C. To dissolve the precipitated formazan salts, DMSO was added to each well for 15 min. The optical density was then measured at 570 nm with reference to 630 nm by using a FLUOstar Omega-microplate reader (BMG Labtech, Cary, NC, USA) [39,40].

### 2.12 *In-vitro* scratch assay

To test the migration ability of the control and treated hA-MSCs and Huh-7 cells, 50,000 cells/well were cultured in 6-well plates (Corning) and incubated at 37 °C and 5 % CO_2_ till reaching 80 % confluency. To exclude that the closure of the scratch is due to the proliferation of the cells, cells were serum starved before scratch induction. Scratch was made using a 10-μl pipette tip, then cells were washed with PBS to remove cell debris. Cells were kept in serum-depleted high glucose DMEM for Huh-7 and serum-depleted low glucose DMEM for hA-MSCs. Using ultrafine tip markings, reference points were made. These reference points were photographed using phase-contrast microscopy at 0, 24, and 48 h. To calculate the migration rate, the gap distance between the cells was measured using ImageJ 1.44P (U.S. National Institutes of Health, Bethesda, Maryland, USA) [41].

### 2.13 Chick chorioallantoic membrane (CAM) assay

To evaluate the angiogenic capacity of normal serum-treated h-AMSCs and HCC serum-treated hA-MSCs, a CAM assay was conducted [42]. Briefly, pathogen-free 5 days post fertilization Egyptian Fayoumi eggs were brought from the integrated Poultry Center (El-Azab, El-Fayoum, Egypt) and incubated in a well-humidified 37 °C egg incubator till the day of the experiment. When the embryos reached the 7.5E stage, a window was made on the air-sac area under aseptic conditions [42]. A sterile cryovial silicon ring was placed on an avascular area of the CAM blood vessels using sterile forceps. About 8 × 10^5^ cells from each group were suspended in 150 μl of sterile egg white albumin, and 50 μl sterile PBS. Cells were mixed well with the diluted albumin, then inoculated inside the silicone ring. The window was sealed using cling film and the eggs were left for 2–5 min for cell settling. The eggs were then carefully placed back again into the egg incubator. On day 12, the CAM membrane was harvested using sterile forceps and the chick embryo was sacrificed directly after that. Two ml of water was then added to each well of a 6-well plate (2 wells for each group) and the isolated membranes were washed atop the water. The membranes were then stored at 4 °C after immersing in PBS.

### 2.14 Protein expression analysis of inflammatory markers using ELISA

Cell culture supernatant was collected in triplicates from normal serum-treated hA-MSCs and Huh-7 and HCC serum-treated hA-MSCs and Huh-7 cells. Samples were centrifuged for 20 min at 1000×*g* and stored at −20 °C until usage. Human MCP-1/CCL2 PicoKine™ kit (MBS175841), human IL-8 PicoKine™ kit (MBS175803), and human TIMP-1 (Tissue Inhibitor of MetalloProteinases-1) kit (E-EL-H0184) were used to analyze the protein level of *CCL2*, *IL8*, and *TIMP-1* according to the manufacturer protocol. The color developed and optical densities (OD) were measured at 450 nm, and the results were normalized against the media used (CCM supplemented with either normal or HCC serum) to exclude serum protein readings from calculations.

### 2.15 Prediction of the interaction between HCC serum proteins and hA-MSCs up-regulated genes

#### 2.15.1 Pipeline for the identification of the differentially expressed proteins

To compare the different experimental conditions in each dataset and obtain a list of differentially expressed genes (DEGs) among HCC serum vs. normal serum; hA-MSCs vs. dermal fibroblasts; and Huh-7 cells cultured in human serum compared to Huh-7 cells cultured in fetal bovine serum (FBS), microarray data were analyzed using the GEO2R online tool. To extract the DEGs and gene expression signatures from GEO datasets, the GEO2Enricher chrome extension was used [35]. To identify the DEGs, both the characteristic direction as well as the *t*-test methods were used. Adjusted p-values were calculated using the Benjamini-Hochberg FDR correction. Detailed methodology is included in Supplementary Material and Methods.

#### 2.15.2 Pipeline for the identification of HCC-secreted proteins

To evaluate the interactions between HCC-secreted proteins and up-regulated genes in hA-MSCs, we identified the up-regulated genes in HCC transcriptome and proteome compared to normal liver, identified these proteins using experimentally validated dataset, and integerated HCC secretome datasets. Detailed methodology is included in Supplementary Material and Methods.

### 2.16 Pipeline for the identification of up-regulated genes in hA-MSCs

To construct a protein-protein interaction (PPI) network between HCC serum proteins and hA-MSCs up-regulated proteins, a complete profile of up-regulated genes in hA-MSCs should be assembled.

Differentially expressed genes sets for hA-MSCs were collected from GEO datasets and Dessels et al., up-regulated genes were combined to yield a total of 1952 genes. Enriched, enhanced, group enriched genes were selected yielding a total of 505 genes. For each gene, the normalized expression values across all the cells were added, and the total sum was scaled and then ranked based on the z-score. Genes having z-score <0 were excluded. Finally, an overlapping analysis was conducted on these gene sets, and theidentified genes were selected for functional enrichment followed by PPI analysis. Detailed methodology is described in the Supplementary Material and Methods.

### 2.17 Functional enrichment analysis

Functional enrichment was performed on both predicted HCC serum proteins, and hA-MSCs up-regulated genes using Enrichr web server (https://amp.pharm.mssm.edu/Enrichr/) [43,44]. Enrichr was used to validate the predicted gene list for HCC serum and hA-MSCs genes in terms of enriched diseases, cell lines, and tissue in addition to pathway annotation and enrichment. For each gene list submitted to Enrichr, the top 10 enriched terms in each library are displayed as a bar graph and ranked according to an adjusted p-value based on the fisher-exact test. An adjusted p-value of <0.05 was considered to indicate a statistically significant result.

### 2.18 Construction of PPI

Interactions between HCC serum proteins and hA-MSCs up-regulated genes were imported from the General Repository for Interaction Datasets (BioGRID) which is an open-access database for the curation and storage of protein, genetic and chemical interactions in major model organisms [45,46]. The BioGRID database (3.5.178) was downloaded and only direct interactions between HCC serum proteins and hA-MSCs genes were filtered. Interactions with other proteins, self-interactions in both datasets, and proteins without connections were excluded. To visualize the molecular interaction, PPI network was drawn using Cytoscape (version 3.5.7) [47]. The degree of interactions between proteins is represented by the size of the nodes. Different groups are represented by different colors and shapes.

### 2.19 Statistical analysis

Gene expression qPCR results were analyzed using a student t-test and Mann–Whitney *U* test. Experimental data are expressed as mean ± standard error of the mean (SEM) of at least three independent experiments. The significant differences between control and experimental groups were identified using one-way analysis of variance (ANOVA) for normally distributed data and Kruskal – Wallis test for non-normally distributed data. P < 0.05 is considered statistically significant. Bonferroni was used as a post-hoc test.

## 3 Results

### 3.1 Changes in the morphology of hA-MSCs and Huh-7 cells upon treatment with normal and HCC serum

**Fig. (1):**
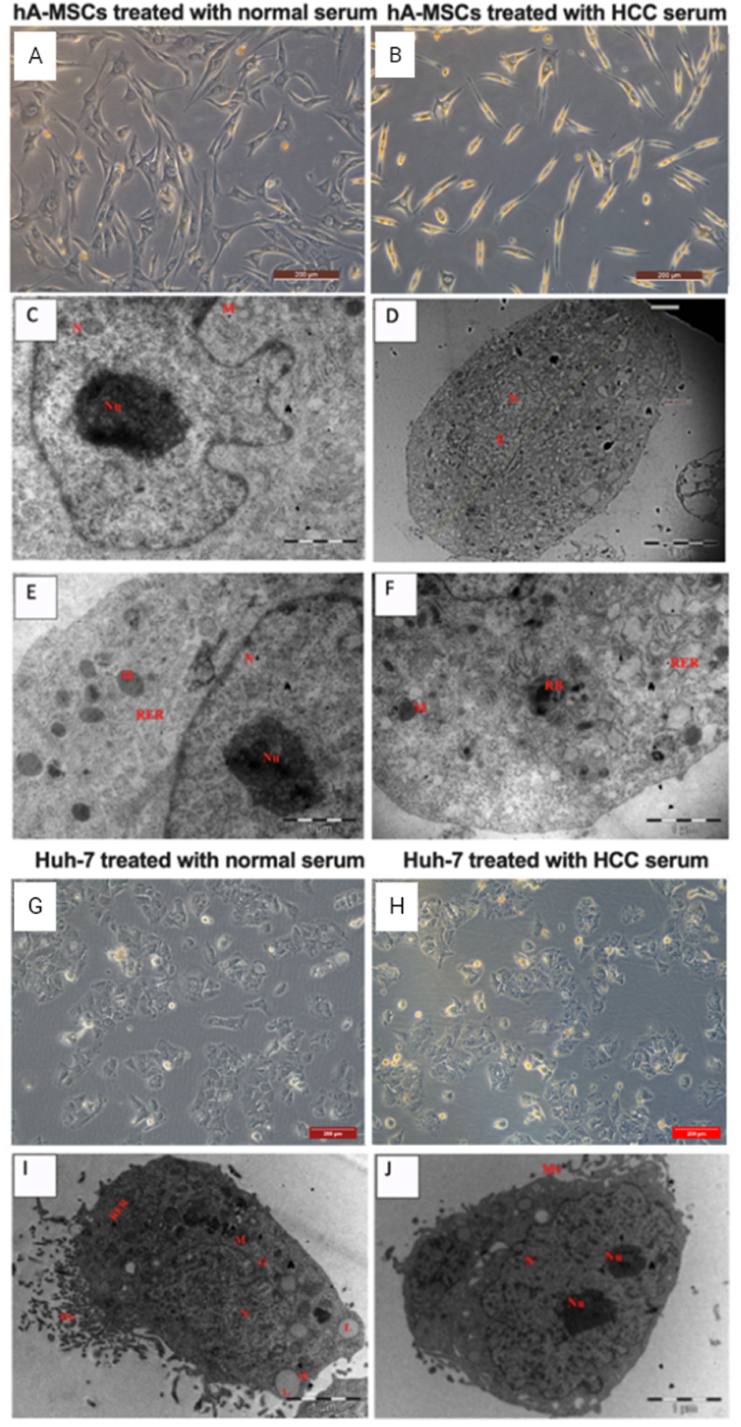
Morphological changes of hA-MSCs and Huh-7 cells after 6 days of culture. Using an inverted fluorescence microscope, the morphology of hA-MSCs **(A and B)** and Huh-7 cells **(G and H)** cultured in normal and HCC serum respectively was assessed. Using a transmission electron microscope, the ultrastructure assessment of hA-MSCs supplemented with normal serum **(C, E)** and HCC serum **(D, F)** took place. **(I, J)** An electron micrograph of Huh-7 cells treated with normal serum and HCC serum respectively for 6 days. Nucleus (N), lipid droplets (L), mitochondria (M), rough endoplasmic reticulum (RER), Golgi apparatus (G), nucleoli (Nu), microvilli (Mv).

Normal serum-treated hA-MSCs for 6 days showed a uniform fibroblast-like spindle shape, with a prominant sphrical nucleus, clear neucleolus and numerous cytoplasmic processes. They have irregular periphery, predominant euchromatin in nuclei, and oval nucleoli as depicted by the transmission electron microscope (TEM) (Fig. 1 C). The cells displayed prominent mitochondria cisternae, rough endoplasmic reticulum (RER) with swollen cisternae, and dark residual cytoplasmic bodies (Fig. 1 E).

HA-MSCs treated with HCC serum for 6 days appeared smaller in size with a smaller nucleus and fewer cytoplasmic processes compared to normal serum-treated hA-MSCs (Fig. 1 A and B). They were oval in shape with irregular periphery, prominent euchromatin, and small nuclei as depicted by the transmission electron microscope. Additionally, organelles were prominent in the cytoplasm, including small mitochondria and RER with swollen cisternae (Fig. 1 D and F). Flow cytometry analysis showed no significant difference in the expression of the surface markers *CD90*, *CD73*, and *CD45* between normal serum-treated and HCC serum-treated hA-MSCs (Fig. S1).

Huh-7 cells treated with normal serum and HCC serum for 6 days showed no change in cell morphology (Fig. 1 G and H). They exhibited typical cancerous features; the cells were irregular in shape with a high density of surface microvilli on the apical cytoplasmic membrane. Their nuclei were irregular with predominant euchromatin and large amounts of heterochromatin, with numerous mitochondria within the cytoplasm and very few rounded lipid droplets (LDs) (Fig. 1 I).

Huh-7 cells treated with HCC serum for 6 days showed features of typical newly dividing oval-shaped cancer cells with visible cytokinesis (Fig. 1 J, S2). The cells had irregularly-shaped nuclei with two nucleoli in each nucleus (Fig. S2 B). They possessed many projecting microvilli, multiple active Golgi apparatus, and a few LDs. Few elongated processes were also observed (Fig. S2 A, B, C).

### 3.2 Changes in the proliferative and apoptotic potential of hA-MSCs and Huh-7 cells upon treatment with normal and HCC serum

**Fig. (2):**
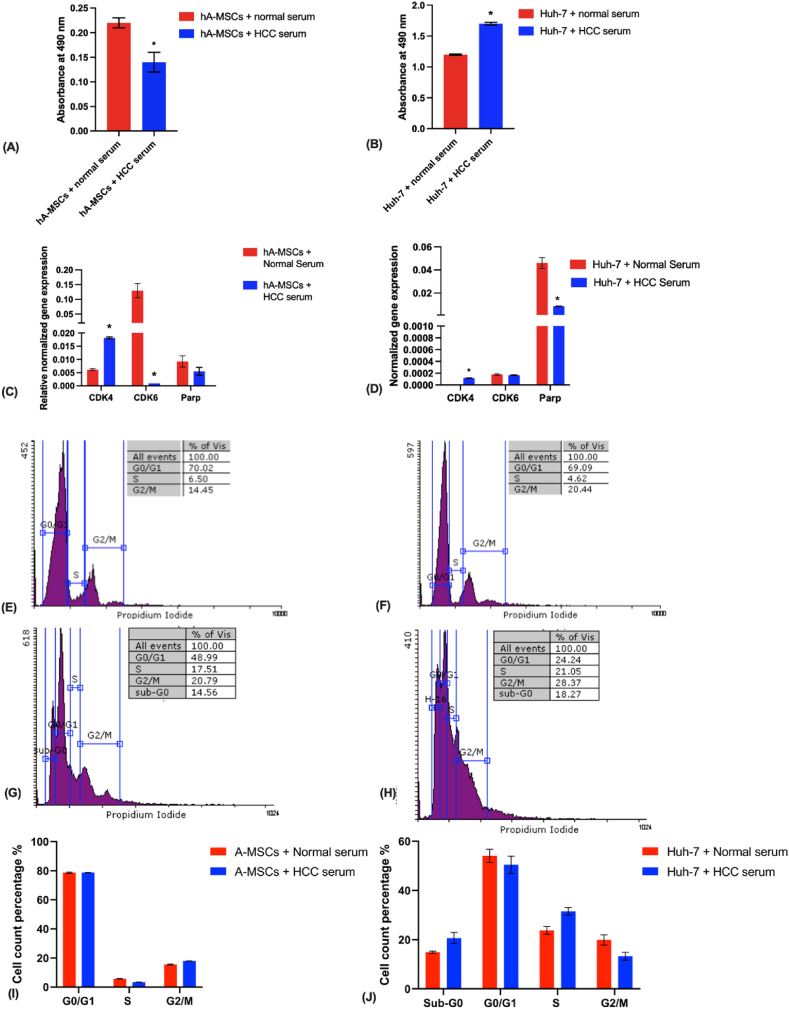
Effect of HCC serum treatment for 6 days on the proliferative capacity of hA-MSCs and Huh-7 cells. (A, B) MTT assay for hA-MSCs and Huh-7 respectively, an indicator of the metabolic activity of these cells. **(C, D)** The normalized expression of proliferation and cell cycle regulators markers in hA-MSCs and Huh-7 respectively assessed by real-time PCR. **(E**–**H)** Assessment of cell cycle progression by flow cytometry. Experiments were repeated at least twice and 10,000 events were analyzed. **(E, F)** The percentage of hA-MSCs in each phase of the cell cycle after treatment with normal and HCC serum cells respectively. **(G, H)** The percentage of Huh-7 cells in each phase of the cell cycle after treatment with normal and HCC serum respectively. **(I, J)** Graphical presentation of the percentage population in G_0_/G_1_, S, and G_2_/M phases for hA-MSCs and sub-G0, G_0_/G_1_, S, and G_2_/M phases for Huh-7 respectively.

**Fig. (3):**
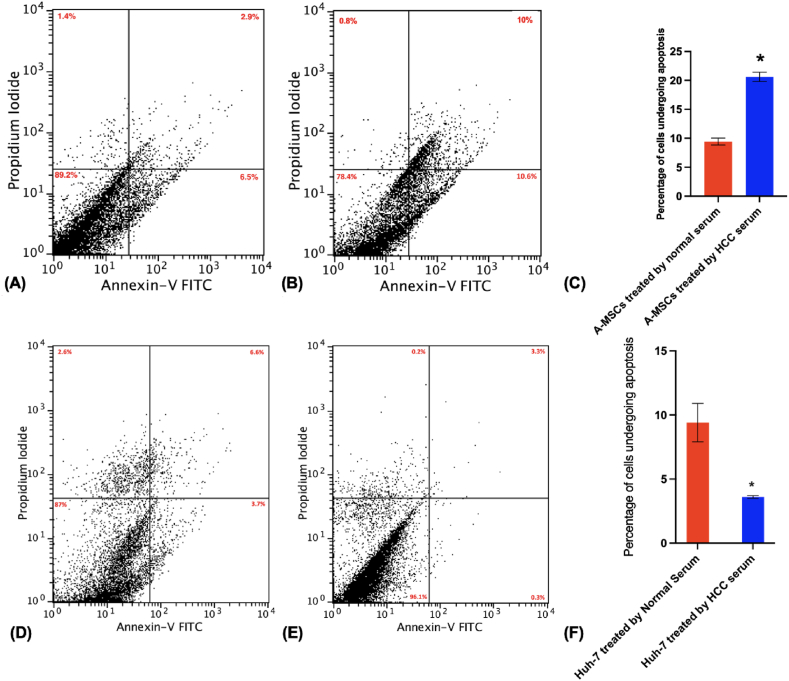
Effect of HCC serum treatment for 6 days on the apoptotic potential of hA-MSCs and Huh-7 cells. (A, B) The percentage of viable, early apoptotic, late apoptotic, and necrotic hA-MSCs treated with normal or HCC serum, **(D, E)** The percentage of viable, early apoptotic, late apoptotic, and necrotic Huh-7 treated with normal or HCC serum. **(C, F)** Bar chart representation of the percentage of control and treated hA-MSCs and Huh-7 undergoing apoptosis respectively.

HCC serum-treated hA-MSCs showed a significant decrease in their cellular proliferation as shown by MTT assay at day 6 (Fig. 2 A). Upon analyzing the cell cycle profile, there was no significant difference in the number of cells in each phase of the cell cycle (Fig. 2 E, F, I). This was accompanied by the downregulation of the cell cycle regulators gene *CDK6*, and the upregulation of the cell cycle regulator *CDK4* (Fig. 2C). Moreover, there was a higher number of cells in the early and late apoptotic phases, a lower number of viable and necrotic cells, and a significant increase in the percentage of cells undergoing apoptosis (Fig. 3A–C).

Huh-7 cells treated with HCC serum for 6 days showed a significant increase in cellular proliferation as shown by MTT proliferation assay (Fig. 2 B), and the significant up-regulation of CDK4 expression (Fig. 2 D). Cell cycle analysis showed no significant difference in the number of cells in each phase of the cell cycle in HCC serum-treated Huh-7 group compared to those treated with normal serum (Fig. 2 G, H, J). This was concomitant with the down-regulation of the DNA repair enzyme PARP-1 (Fig. 2 D). Moreover, there was a lower number of cells in early and late apoptotic phases, and a higher number of viable cells compared to the normal serum-treated Huh-7 cell group (Fig. 3D–F).

### 3.3 Changes in the migration potential and the cancer progression of hA-MSCs and Huh-7 cells upon treatment with normal and HCC serum

**Fig. (4):**
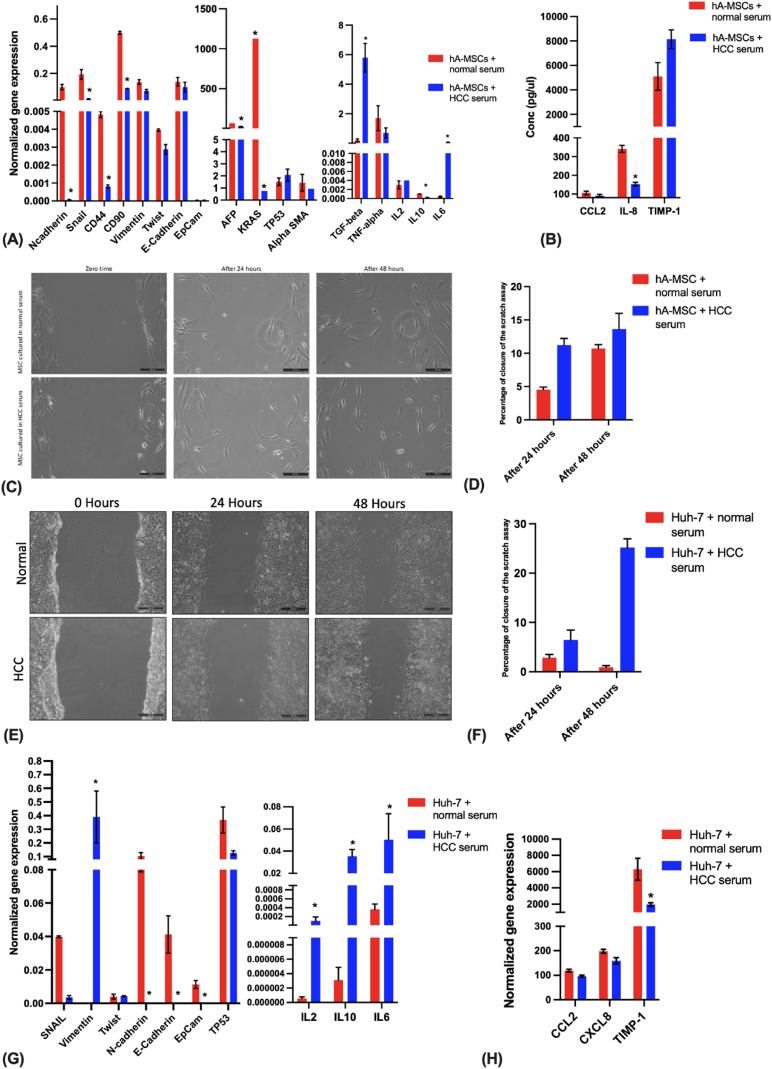
Effect of HCC serum treatment for 6 days on the migration potential and cancer progression of hA-MSCs and Huh-7 cells. (A, G) Normalized gene expression analysis using real-time PCR in normal and HCC serum-treated hA-MSCs and Huh-7 respectively. **(B, H)** Protein expression of CCL2, IL8, and TIMP-1 assessed by Enzyme-linked immunosorbent assay (ELISA) in normal and HCC serum-treated hA-MSCs and Huh-7respectively. **(C, E)** Scratch assay for normal and HCC serum-treated hA-MSCs and Huh-7 respectively. This was assessed at zero time, 24 h, and 48 h. Pictures were taken using an inverted fluorescence microscope and scale bars (200 μm) were added. **(D, F)** Bar chart representation of the percentage of closure of the scratch in normal and HCC serum-treated hA-MSCs and Huh-7 respectively.

HCC serum-treated hA-MSCs for 6 days showed no significant change in the migration potential (Fig. 4 C, D). This was accompanied by a downregulation in the mesenchymal markers (*N-Cadherin*, *SNAIL*, *and CD90*) and the mesenchymal adhesion marker (*CD44*) (Fig. 4 A). While the epithelial markers *E-Cadherin* and *Epcam* did not significantly change (Fig. 4 A). There was also a significant downregulation of the oncogene *KRAS*, the anti-inflammatory marker *IL-10*, and the liver-specific gene alpha-fetoprotein (*AFP*), also considered as an HCC biomarker (Fig. 4 A). This is concomitant with the downregulation of the pro-inflammatory marker *IL-8* on the protein level (Fig. 4 B). Moreover, upon prolonging the treatment to 21 days, hA-MSCs showed a decrease in their migration potential (Fig. S3 B).

HCC serum-treated Huh-7 cells showed a significant increase in the migration capacity compared to normal serum-treated Huh-7 cells as shown by the scratch assay (Fig. 4 E, F). This was accompanied by the upregulation of the mesenchymal marker *Vimentin* and the concomitant downregulation of the epithelial markers (*E-cadherin* and *Epcam*) (Fig. 4 G). This was also accompanied by an upregulation of hepatocarcinogenesis-related cytokines *IL-2*, *IL-10*, and *IL-6* (Fig. 4 G). This was further confirmed by the significant down-regulation of *TIMP-1* on the protein level upon treatment (Fig. 4H). However, the expression of the mesenchymal markers *SNAIL* and *N-Cadherin* decreased significantly upon treatment with HCC serum for 6 days compared to that of normal serum-treated cells (Fig. 4 G).

### 3.4 Changes in the pluripotency and differentiation potential of hA-MSCs and Huh-7 cells upon treatment with normal and HCC serum

**Fig. (5):**
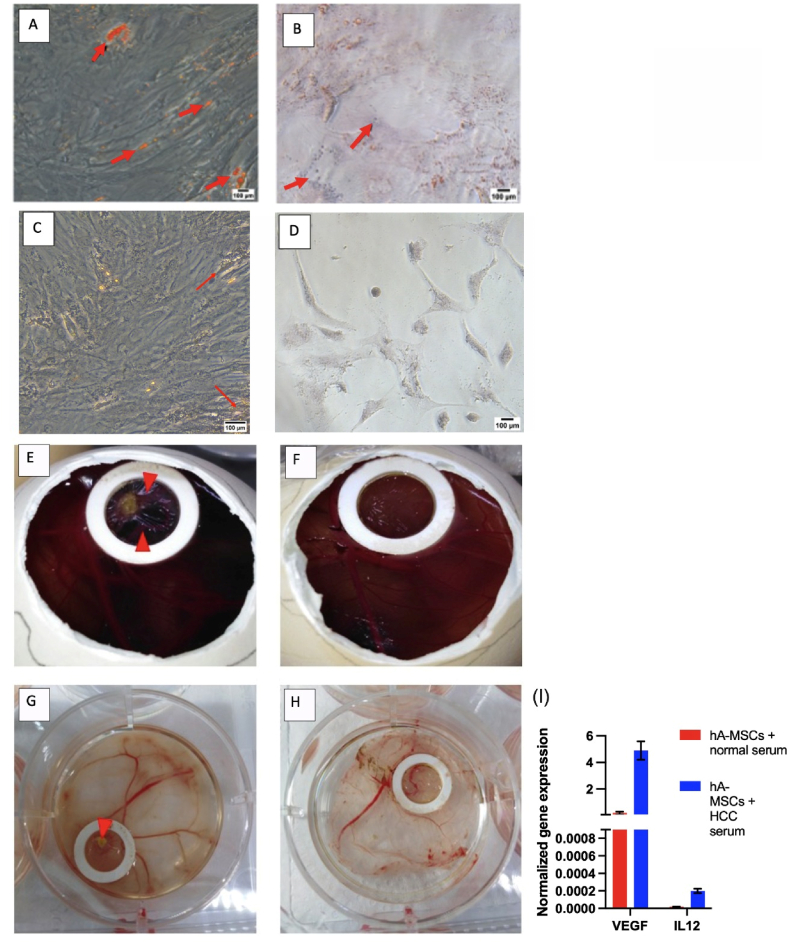
Effect of HCC serum treatment for 6 days on the adipogenic, osteogenic, and angiogenic potential of hA-MSCs. (A, B) The adipogenic differentiation potential was observed in normal serum-treated hA-MSCs (A) compared to HCC serum (B). Arrow indicates the formation of Oil red O-stained oil droplets in response to adipogenic induction. **(C,D)** The osteogenic differentiation potential was observed in normal serum treated-hA-MSCs (C) compared to HCC serum (D). Arrow indicates the formation of orange-stained Ca^2+^ deposits in response to osteogenic induction. **(E**–**H)** CAM assay for evaluating the angiogenesis *in-ovo* 5 days post-implantation in **(E)** Normal serum-treated hA-MSCs, **(F)** HCC serum-treated hA-MSCs. **(G**–**H)** Isolated CAM from the egg with **(G)** Normal serum-treated hA-MSCs, **(H)** HCC serum-treated hA-MSCs. **(I)** Normalized gene expression analysis using real-time PCR in normal and HCC serum-treated hA-MSCs. (For interpretation of the references to color in this figure legend, the reader is referred to the Web version of this article.)

**Fig. (6):**
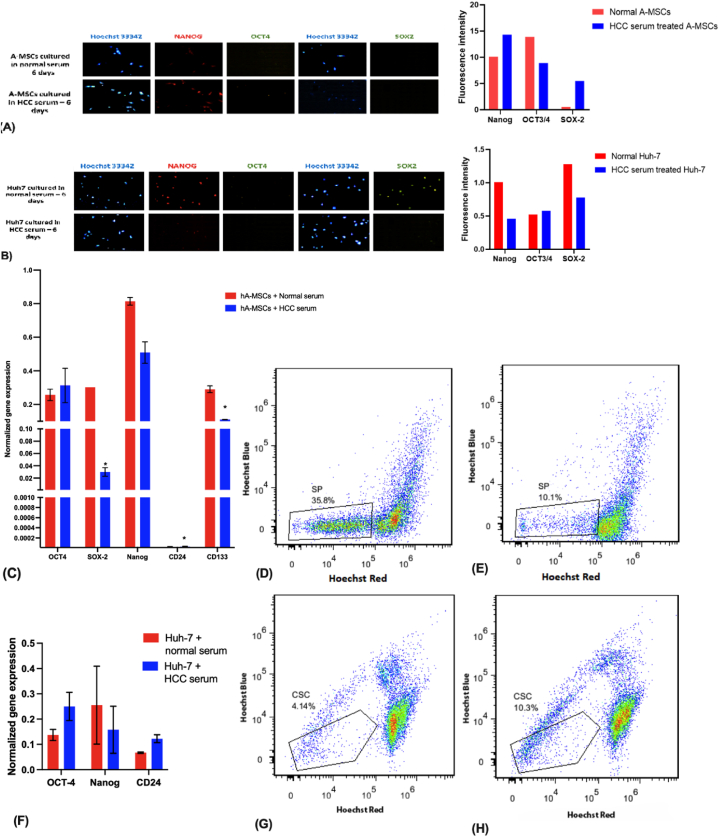
Effect of serum from HCC patients on the pluripotency and chemoresistance of hA-MSCs and Huh-7 cells after 6 days of treatment. (A, B) Expression of OCT-4, SOX-2, and NANOG pluripotency proteins using immunofluorescence technique in **(A)** hA-MSCs cultured in normal serum and HCC serum, **(B)** Huh-7 cells cultured in normal serum and HCC serum. **(C, F)** Gene expression analysis using real-time PCR in normal and HCC serum-treated hA-MSCs and Huh-7 cells respectively. **(D**–**H)** Side population (SP) assay for normal and HCC serum-treated hA-MSCs (D,E) and normal and HCC serum-treated Huh-7 (G, H) respectively.

HCC serum-treated hA-MSCs showed a decrease in the adipogenic and osteogenic differentiation in HCC serum-treated hA-MSCs compared to normal serum-treated group. This was shown by the decrease in Oil red O-stained oil droplets and orange-stained Ca^2+^ deposits respectively (Fig. 5A–D). There was also a decrease in the angiogenic capacities of these cells. Normal serum-treated hA-MSCs showed small cellular mass surrounded by small blood vessels while there was no change in the cellularity or blood vessel structure in HCC serum-treated hA-MSCs (Fig. 5E–H) [64,65]. This was confirmed by the upregulation of the anti-angiogenic marker *IL-12*. However, there was also an up-regulation in the angiogenic marker (*VEGF*) in the hA-MSCs treated with HCC serum for 6 days (Fig. 5 I). This is concomitant with a decrease in the pluripotency marker *SOX-2*, while no significant change was observed in *OCT-4* and *Nanog* (Fig. 6 A and C). HCC serum-treated Huh-7 cells showed no significant difference in the pluripotency markers *SOX-2, OCT-4 and Nanog* (Fig. 6 B and F).

### 3.5 Changes in the chemoresistance of hA-MSCs and Huh-7 cells upon treatment with normal and HCC serum

HCC serum-treated hA-MSCs showed a reduction in the chemoresistance as shown by the decreased percentage of cells showing side population (SP) properties, concomitant with a decrease in the expression of cancer stem cell marker *CD133* in HCC serum-treated hA-MSCs (Fig. 6C, D and E). HCC serum-treated Huh-7 cells showed an induction of chemoresistance, as shown by the increase in the percentage of cells showing side population properties (Fig. 6 G and H). This was concomitant with a significant up-regulation of HCC maintenance and self-renewal marker, *CD24* (Fig. 6 F).

### 3.6 Interaction of HCC serum proteins with hA-MSCs

**Fig. (7):**
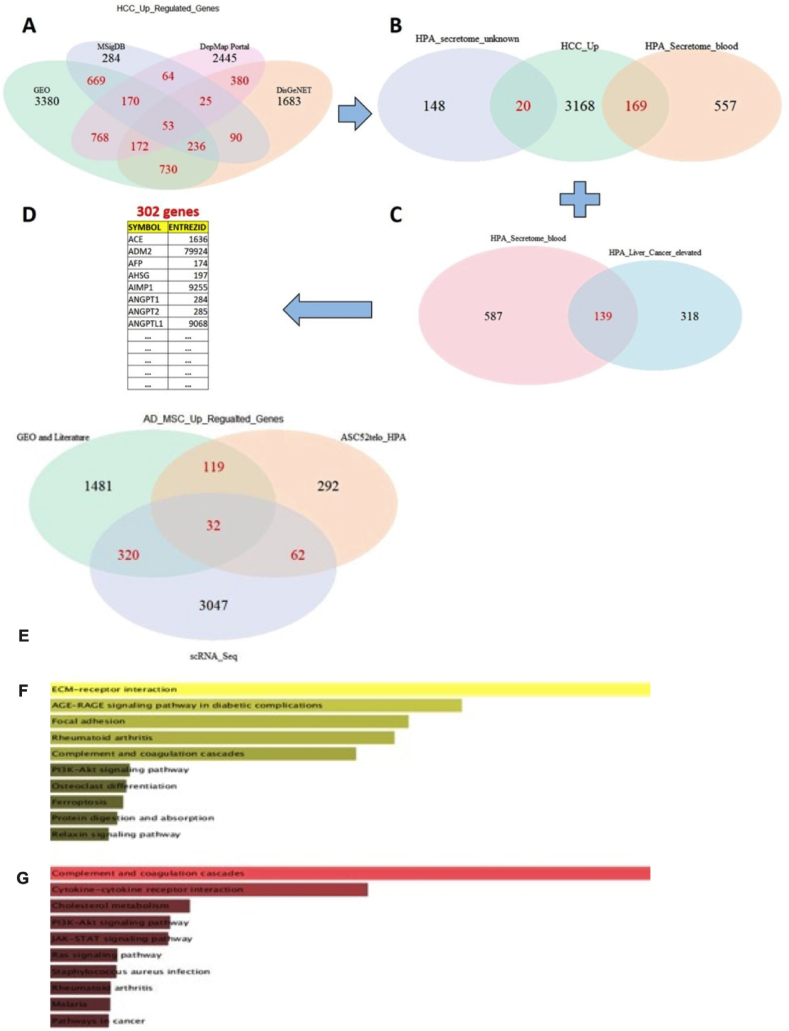
Identification of the secreted proteins in HCC serum. Venn diagrams of **(A)** The up-regulated gene sets in HCC compared to the normal liver were obtained from four databases and repositories. **(B)** An overlapping analysis was performed to identify the proteins that are upregulated in HCC from the previous step that overlaps with HPA human secretome. **(C)** An overlapping analysis was performed to identify the proteins that are found to be highly expressed in liver cancer according to the pathology atlas in HPA which are also found to be secreted according to the HPA secretome. **(D)** Combining the proteins from (B) and (C) yielded a total of 302 proteins (after removing duplicates), which were selected for the functional enrichment followed by protein-protein interaction (PPI) analysis. **(E)** The identification of up-regulated genes in hA-MSCs. Venn diagram of an overlapping analysis conducted on the up-regulated gene sets in hA-MSCs. These genes were selected for functional enrichment followed by PPI analysis. **(F,G)** KEGG pathway enrichment analysis. **(F)** Bar graph displaying the top 10 enriched KEGG pathways for the A-MSCs gene set comprising 533 genes. **(G)** Bar graph displaying the top 10 enriched KEGG pathways for the HCC secretome gene set comprising 302 genes. The bars are ranked according to adjusted p-value based on the fisher-exact test. **GEO**: Gene Expression Omnibus; **MsigDB**: Molecular Signature Database; **DepMap portal**: Dependency map portal; **DisGeNET**: Disease Gene Network database; **HPA**: Human Protein Atlas; **HCC**: Hepatocellular Carcinoma; **GEO**: Gene Expression Omnibus; **ASC52telo_HPA**: telomerase immortalized hA-MSCs cell line; **scRNA_Seq**: single-cell RNA-Seq of hA-MSCs; **A-MSCs**: Adipose-derived mesenchymal stromal cells; **PPI**: Protein-protein Interaction; **HCC**: Hepatocellular carcinoma.

A complete list of overexpressed proteins found in HCC serum has been identified in this study and can be found in Dataset S2. Comprehensive bioinformatics analysis revealed a total of 302 proteins identified as a representative gene signatures of HCC secretome in the serum (Fig. 7A–D).

Further enrichment analysis of the gene sets revealed that the identified genes were enriched in HCC cell lines, including HepG2, Hep3B, and Huh-7 according to the cancer cell line encyclopedia (CCLE), NCI-60 dataset and ARCHS4 curated RNA-Seq database (Fig. S4 A-C).

Enriched GO terms involved cytokine, chemokine, and growth factor as molecular functions, pathway terms including platelet degranulation, cytokine-mediated signaling, and regulation of complement activation.

The predicted proteins were also enriched in compartments including extracellular space, blood microparticles, and extracellular exosomes according to the Jensen compartments database (Fig. S4 D-F).

KEGG pathway enrichment analysis revealed immune response and inflammation-related genes and pathways. The top enriched pathways were complement and coagulation cascades, cytokine-cytokine receptor interaction, and JAK-STAT signaling pathways.

**Fig. (8):**
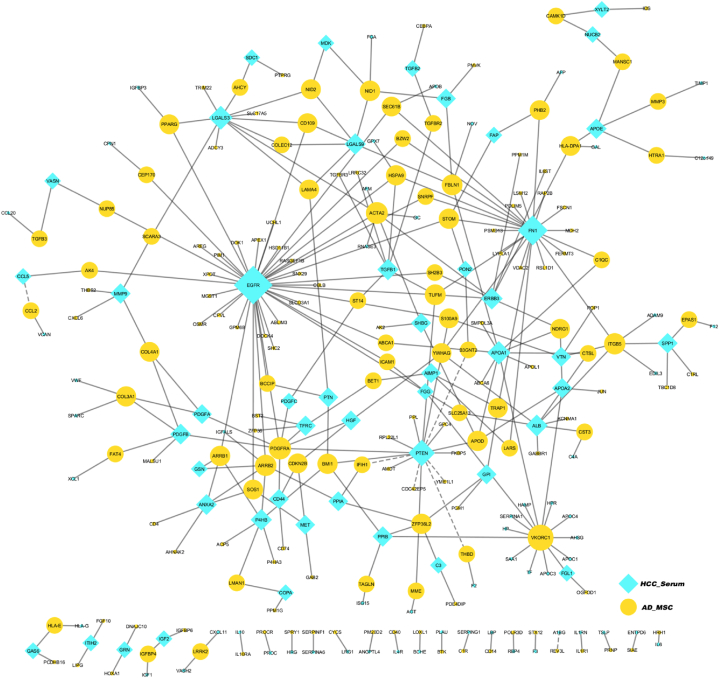
Protein-Protein interaction (PPI) interaction between HCC serum proteins and hA-MSCs. The PPI interaction network displays undirected interactions between predicted proteins in the HCC secretome and predicted overexpressed proteins in A-MSCs based on data obtained from the BioGrid database. The size of the nodes represents the degree (the number of interactions with other proteins). Solid edges represent physical interactions, while dashed edges represent genetic interactions. HCC secretome proteins are illustrated as blue diamond nodes, and A-MSCs proteins are represented as circular yellow nodes. **A-MSCs**: Adipose-derived mesenchymal stromal cells; **HCC**: Hepatocellular carcinoma. (For interpretation of the references to color in this figure legend, the reader is referred to the Web version of this article.)

Regarding hA-MSCs, a total of 533 genes were identified as representative gene signatures of hA-MSCs (Fig. 7 E). KEGG enrichment analysis revealed cell adhesion and migration-related genes and pathways. The top enriched pathways were ECM-receptor interaction, focal adhesion, and AGE-RAGE signaling (Fig. 7 F, G). Finally, an undirected protein-protein interaction network (PPI) was constructed using data from BioGrid [51,52] to visualize the predicted physical and genetic interactions between the predicted proteins in the HCC serum and the top-upregulated genes in hA-MSCs (Fig. 8). The network shows EGFR, PTEN, and FN1 as hub proteins in HCC serum through which multiple interactions are mediated.

## 4 Discussion

The applications of the migration ability of MSCs to the tumor site was exploited in tumor-targeted therapy [48]. Recent reports showed that hA-MSCs-based cell therapy could be promising for replacing, repairing, and regenerating damaged cells at the tumor site [49]. Despite favorable pre-clinical data, Seo et al. reported that h-AMSCs showed limited efficacy and low expectations in advanced clinical trials [38]. Little data are available on the crosstalk between the MSCs at the tumor site and their surrounding cells, and how the tumor microenvironment impacts the transplanted cells [49]. To exert their desired therapeutic potency, hA-MSCs should survive long enough, proliferate, migrate effectively to the site of injury, and perform their function of interest in tissue regeneration. These functions include angiogenesis, appropriate proliferation, differentiation, and immunomodulation [4,[13], [14], [15]]. To study the potential of applying effective stem cell-based therapy in HCC, we investigated the direct impact of serum from HCC patients on the regenerative capacities of hA-MSCs [50].

Through comprehensive bioinformatic analysis, 302 proteins were found in the HCC serum. The enriched pathways influence the immune and inflammatory response concurrent with HCC development and progression to promote tumor growth and immunosuppression [[51], [52], [53]].

We investigated the effect of serum from HCC patients on maintaining cancer properties in liver cancer cells. We showed that upon treatment of Huh-7 by HCC serum, there was a higher tumor aggressiveness. This was proved by the change in morphology, the increase in the proliferation, migration, and the decrease in the percentage of cells undergoing early and late apoptosis, and the presence of lipid droplets [54]. Lipid droplets (LDs) are required for cell survival, proliferation, angiogenesis, and metastasis to sustain the high energy consumption [[55], [56], [57], [58], [59]]. Higher tumor aggressiveness was also shown by the upregulation of the anti-inflammatory markers, *IL-6* and *IL-10*, the upregulation of the proinflammatory marker *Il-2*, and the downregulation of *TP53* and *TIMP-1* [59]. *IL-6* increases Huh-7 cell migration and enhances cancer stemness [60]. *IL-10* is correlated with cancer progression and metastasis [61]. *IL-2* is involved in escaping the immune response [59]. *TP53* is a tumor suppressor marker [62]. *TIMP-1* inhibits tumor cell proliferation, migration, angiogenesis, and apoptosis [63].

These findings were concomitant with significant upregulation in the cancer stemness marker *CD24* expression. *CD24*^*+*^ HCC cells were important for maintenance, self-renewal, differentiation, metastasis of tumors, induction of chemoresistance, and significantly influenced patients’ clinical outcomes [62]. An increase in chemoresistance properties was shown by the increase in the percentage of cells displaying SP properties and by the presence of LDs [[64], [65], [66], [67]].

When the treatment duration of Huh-7 cells was prolonged to 21 days, there was an upregulation of the mesenchymal markers and a down-regulation of the epithelial marker, which shed the light on the possibility of the activation of the epithelial to the mesenchymal transition process. This was accompanied by the upregulation of the pluripotency genes, *OCT-4*, *SOX-2*, and *NANOG*, which points to a possible increase in stemness [68,69].

When we investigated the effect of HCC serum on hA-MSCs, our data showed that high levels or activity of oncogenic proteins in serum from HCC patients induced oncogene-induced senescence of hA-MSCs [38]. This was shown by the reduced proliferation, the reduced adipogenic, osteogenic, and angiogenic differentiation capacity, and the altered morphology of HCC-serum-treated h-AMCs. Previous reports showed that the application of cancer sera on hESCs inhibited stem cell differentiation and supported their transformation into CSCs [11,70]. The decreased angiogenic potential was confirmed by the downregulation of angiogenic markers (*VEGF*, *HGF*, and *CD146*), and by the absence of neovascularization and cellular masses in an *in-ovo* model chick CAM [10,11,38,67,[70], [71], [72], [73], [74], [75], [76], [77], [78], [79], [80], [81], [82]]. However, these cells did not transform into CSCs as shown by the decrease in cells displaying SP properties and the downregulation of the CSC marker *CD44* in HCC-treated hA-MSCs [80,81,83].

Oncogene-induced senescence was also shown by the abnormal fibroblast morphology, their enlarged appearance, the small nucleus with the irregular periphery, the more swollen cisternae of rough endoplasmic reticulum, and the presence of dark residual bodies, which represent aged lysosomes. These lysosomes with absent digesting capacity indicate cellular stress as shown by the appearance of indigestible residues inside the cell [13].

This is also accompanied by an increase in the percentage of cells in the early and late apoptosis phases in the HCC serum-treated hA-MSCs compared to normal serum-treated hA-MSCs. A decrease in the level of the pro-inflammatory marker *TNF-α* was also observed, which suppresses the inflammatory milieu and reduces the anti-inflammatory properties of h-AMCs [80,81,84,85]. Downregulation of the anti-inflammatory marker *IL-10*, and the pro-inflammatory markers *IL-12* and *IL-8*. *IL-10* was found to promote proliferation and migration and inhibit differentiation in MSCs [15]. *IL-12* inhibition is involved in escaping the immune response [86,87]. This is aligned with reported data that most systemically administered MSCs disappeared after 24 h, and less than 1 % of the cells survived more than a week [88].

When the treatment duration of hA-MSCs was prolonged to 21 days, there was a decrease in their migration potential and the expression of the adhesion and migration marker *CD44*. This might affect MSC migration via the binding of cell surface adhesion molecule *CD44* to hyaluronic acid (HA) of the ECM. The enriched pathways in the hA-MSCs provided evidence for increased cell migration, which can be partially attributed to the intrinsic homing property and recruitment of hA-MSCs to the tumor site mediated by released inflammatory cytokines such as *IL6* and *TNF-α* from tumor cells [89]. Collectively, these results provide a potential framework delineating the crosstalk between HCC serum proteins and hA-MSCs. HCC serum comprises a wide range of different proteins that we identified via proteomic analysis and were found to affect the biological properties of MSCs. One of the important differentially expressed sets of proteins was found to be connected with ERK1/2 and nuclear factor-κB (NF-κB) signaling pathways, besides MMPs up-regulation [90]. It was reported that the OPN-activated FAK/ERK1/2 pathway is associated with a decrease in the number of the organized actin cytoskeleton and a decrease in the cell stiffness reflected in BM-MSCs-induced migration [91]. However, this potential was later on diminished, which could be attributed to the decrease in the ERK1/2 associated factors [92]. This is in line with our results that showed no change in the migration potential of h-AMSCs after exposure to HCC serum for 6 days, followed by a decrease in migration potential after 21 days.

Our findings provide insights into interactions between MSCs and factors present in the serum of HCC patients, with the potential of finding novel targets for cancer therapy including hA-MSCs.

### 4.1 Study limitations

There are limited data on human serum as an alternative to FBS for the use and expansion of stem cells. Additionally, the effect of human serum or various factors of the HCC microenvironment on the regerative cpacities of MSCs is hindered by the lack of long term *in-vitro* and *in-vivo* data, and the availability and variability of patient serum. Using patient tumor tissue will also significantly enhance the development of stem cells as precision personalized therapy for cancer. Studies using 3-D constrcuts for HCC, including organoids are currently ongoing in our laboratory to provide more accurate and relevant model to study HCC micrornviromnet and its effect on MSCs regenrative capacities.

## 5 Conclusion

Our findings provide preliminary evidence against the regenerative capacity of hA-MSCs mediated by the effects of HCC circulating soluble factors in the serum while maintaining cancer properties in Huh-7 liver cancer cells. This might give insight into the weak functionality, poor survival, and poor migration capabilities of injected h-AMSCs in hosts leading to unsatisfactory clinical results.

## Ethics statement

The protocol for obtaining serum samples was reviewed and approved by the IRB from the Faculty of Medicine Cairo University, Cairo University - Faculty of Medicine - Research Ethics Comittee, protocol approval number: N-56-2020, and the National Liver Institute Monoufia University, NLI IRB 00003413 FWA0000227, protocol approval number: 00140/2018. All participants provided informed consents to participate in the study.

## Funding statement

This work is supported bty grants # 5300 and grant #46721 from the Egyptian Science and 10.13039/100006180Technology Development Fund, Cairo, Egypt and by grant #2019/003 from 10.13039/501100010720Zewail City of Science and Technology, Giza, Egypt.

## Data availability statement

All data generated or analyzed during this study are included in this published article and its additional files.

## CRediT authorship contribution statement

**Radwa Ayman Salah:** Writing – original draft, Validation, Project administration, Methodology, Investigation, Formal analysis, Conceptualization. **Azza M. El-Derby:** Writing – original draft, Methodology, Investigation, Formal analysis. **Zaynab El-Gammal:** Writing – original draft, Validation, Investigation, Formal analysis, Data curation. **Bishoy Wadie:** Writing – original draft, Software, Formal analysis. **Sara M. Ahmed:** Investigation, Formal analysis. **Shimaa E. Elshenawy:** Methodology, Investigation. **Shireen Magdy:** Methodology, Formal analysis, Data curation. **Ayman Salah:** Visualization, Resources, Conceptualization. **Mahmoud Gabr:** Visualization, Resources, Data curation. **Ihab Mohamed:** Visualization, Validation, Methodology, Formal analysis. **Nagwa El-Badri:** Writing – review & editing, Supervision, Resources, Project administration, Methodology, Funding acquisition, Conceptualization.

## Declaration of competing interest

The authors declare that they have no known competing financial interests or personal relationships that could have appeared to influence the work reported in this paper.

The authors declare the following financial interests/personal relationships which may be considered as potential competing interests:Nagwa El-Badri reports financial support was provided by The Egyptian Science and 10.13039/100006180Technology Development Fund. If there are other authors, they declare that they have no known competing financial interests or personal relationships that could have appeared to influence the work reported in this paper.
