# Supplementary material for: Hepatocellular carcinoma patients serum modulates the regenerative capacities of adipose mesenchymal stromal cells
Source: Heliyon. 2024 Feb 1;10(3):e24794. doi: 10.1016/j.heliyon.2024.e24794 (PMC10850426; doi:10.1016/j.heliyon.2024.e24794)
Supplement: Multimedia component 2 [file mmc2.docx]

**Supplementary data**

**Supplementary material and methods**

**Table S1.** Human forward and reverse primer sequences

| **Accession number** | **Primer** | **Sequence** | | **Amplicon length** |
| --- | --- | --- | --- | --- |
| Q01860 | OCT4 | Forward | TGTACTCCTCGGTCCCTTTC | 150 |
|  |  | Reverse | TCCAGGTTTTCTTTCCCTAGC |  |
| P48431 | SOX-2 | Forward | GCTAGTCTCCAAGCGACGAA | 144 |
|  |  | Reverse | GCAAGAAGCCTCTCCTTGAA |  |
| Q9H9S0 | NANOG | Forward | CAGTCTGGACACTGGCTGAA | 149 |
|  |  | Reverse | CTCGCTGATTAGGCTCCAAC |  |
| P08670 | Vimentin | Forward | TGTCCAAATCGATGTGGATGTTTC | 117 |
|  |  | Reverse | TTGTACCATTCTTCTGCCTCCTG |  |
| O95863 | SNAIL | Forward | ACCACTATGCCGCGCTCTT | 115 |
|  |  | Reverse | GGTCGTAGGGCTGCTGGAA |  |
| P12830 | E-Cadherin | Forward | GTCACTGACACCAACGATAATCCT | 99 |
|  |  | Reverse | TTTCAGTGTGGTGATTACGACGTTA |  |
| P19022 | N-Cadherin | Forward | GGTGGAGGAGAAGAAGACCAG | 72 |
|  |  | Reverse | GGCATCAGGCTCCACAGT |  |
| P11802 | CDK4 | Forward | TCGAAAGCCTCTCTTCTGTG | 100 |
|  |  | Reverse | TACATCTCGAGGCCAGTCAT |  |
| Q00534 | CDK6 | Forward | GCTGGTAACTCCTTCCCCAG |  |
|  |  | Reverse | GTCCAGAATCATTGCACCTGAG |  |
| O00716 | E2F3 | Forward | GAGACTGAAACACACAGTCC | 98 |
|  |  | Reverse | CCTGAGTTGGTTGAAGCC |  |
| P01116 | K-RAS | Forward | TGTTCACAAAGGTTTTGTCTCC | 127 |
|  |  | Reverse | CCTTATAATAGTTTCCATTGCCTTG |  |
| Q12888 | TP53 | Forward | GTTCCGAGAGCTGAATGAGG | 123 |
|  |  | Reverse | TTATGGCGGGAGGTAGACTG |  |
| P16422 | EPCAM | Forward | CGCAGCTCAGGAAGAATGTG | 88 |
|  |  | Reverse | TGAAGTACACTGGCATTGACG |  |
| O43490 | CD133 | Forward | CAGAGTACAACGCCAAACCA | 245 |
|  |  | Reverse | AAATCACGATGAGGGTCAGC |  |
| P25063 | CD24 | Forward | TGCTCCTACCCACGCAGATT | 89 |
|  |  | Reverse | GGCCAACCCAGAGTTGGAA |  |
| P16070 | CD44 | Forward | AGAAGGTGTGGGCAGAAGAA | 116 |
|  |  | Reverse | AAATGCACCATTTCCTGAGA |  |
| P05231 | IL-6 | Forward | GTAGCCGCCCCACACAGACAGCC | 173 |
|  |  | Reverse | GCCATCTTTGGAAGGTTC |  |
| P62736 | α-SMA | Forward | CCGACCGAATGCAGAAGGA | 88 |
|  |  | Reverse | ACAGAGTATTTGCGCTCCGAA |  |
| P02771 | AFP | Forward | AGCAGCTTGTTAAATCAACATGCA | 111 |
|  |  | Reverse | AAAATTAACTTTGGTAAACTTCTGACTCAGT |  |
| P60709 | Beta Actin | Forward | AGAGCTACGAGCTGCCTGAC | 184 |
|  |  | Reverse | AGCACTGTGTTGGCGTACAG |  |
| P04216 | CD90 | Forward | TCAGGAAATGGCTTTTCCCA | 101 |
|  |  | Reverse | TCCTCAATGAGATGCCATAAGCT |  |
| P22301 | IL-10 | Forward | GTGATGCCCCAAGCTGAGA | 138 |
|  |  | Reverse | CACGGCCTTGCTCTTGTTTT |  |
| P29459 | IL-12 | Forward | TGGAGTGCCAGGAGGACAGT | 147 |
|  |  | Reverse | TCTTGGGTGGGTCAGGTTTG |  |
| P60568 | IL-2 | Forward | AACTCACCAGGATGCTCACA | 105 |
|  |  | Reverse | GCACTTCCTCCAGAGGTTTGA |  |
| P09874 | PARP | Forward | AGCGTGTTTCTAGGTCGTGG | 194 |
|  |  | Reverse | CATCAAACATGGGCGACTGC |  |
| P01137 | TGF-beta | Forward | CAGCAACAATTCCTGGCGATA | 136 |
|  |  | Reverse | AAGGCGAAAGCCCTCAATTT |  |
| P01375 | TNF-α | Forward | TCTTCTCGAACCCCGAGTGA | 151 |
|  |  | Reverse | CCTCTGATGGCACCACCAG |  |
| Q15672 | Twist | Forward | CGGGAGTCCGCAGTCTTA | 161 |
|  |  | Reverse | GCTTGAGGGTCTGAATCTTG |  |
| P15692 | VEGF | Forward | TGCAGATTATGCGGATCAAACC | 81 |
|  |  | Reverse | TGCATTCACATTTGTTGTGCTGTAG |  |

***Pipeline for the identification of the differentially expressed proteins***

Microarray Gene expression omnibus (GEO) datasets GSE117361, GSE62232, GSE108511, and GSE72332 were downloaded from GEO database [1]. Samples in the aforementioned datasets were divided into three groups: HCC serum vs. normal serum; hA-MSCs vs. dermal fibroblasts (controls used in the selected gene expression data set); and Huh-7 cells cultured in human serum versus Huh-7 cells cultured in fetal bovine serum (FBS). Detailed parameters of each dataset are displayed in (Table S2). To compare the different experimental conditions in each dataset and obtain a list of differentially expressed genes (DEGs) among the defined groups, microarray data were analyzed using the GEO2R online tool. To extract the DEGs and gene expression signatures from GEO datasets, the GEO2Enricher chrome extension was used [2]. To identify the DEGs, both the characteristic direction as well as the t-test methods were used. Adjusted p-values were calculated using the Benjamini-Hochberg FDR correction. An adjusted p-value of <0.05 was considered to indicate a statistically significant difference. Only up-regulated genes in each group relative to the relevant control were selected for downstream analysis.

**Table S2:** Details of datasets from the GEO database used to retrieve the differentially expressed gene sets

| **Author/year** | **GEO dataset** | **Group name** | **No. of samples** | **Treatment group** | **Control group** | **Refs.** |
| --- | --- | --- | --- | --- | --- | --- |
| Yong Jiang, 2019 | GSE117361 | Tumor tissue _HCC | 2 | Tumor tissue | normal tissue liver | [3] |
|  |  | Normal tissue liver | 2 |  |  |  |
| Kornelius Schulze, 2015 | GSE62232 | HCC liver tumor | 81 | HCC liver tumor | Non-tumor liver | [4] |
|  |  | Non-Tumor Liver | 10 |  |  |  |
| Dana Hattab, 2019 | GSE108511 | Bone marrow | 5 | Adipose tissue | Dermal fibroblast | [5] |
|  |  | Adipose tissue | 3 |  |  |  |
|  |  | Umbilical cord | 4 |  |  |  |
|  |  | Placenta | 3 |  |  |  |
|  |  | Dermal fibroblast | 3 |  |  |  |
| Beatriz Roson Burgo, 2016 | GSE72332 | A-MSC | 3 | A-MSCs | Fibroblast | [6] |
|  |  | BM-MSC | 3 |  |  |  |
|  |  | PLMSC | 3 |  |  |  |
|  |  | HSPC | 3 |  |  |  |
|  |  | FIB | 3 |  |  |  |

**A-MSC:** Adipose-derived Mesenchymal Stem Cells; **BM-MSC:** Bone marrow-derived mesenchymal stem cells; **PLMSC:** Mesenchymal Stem/Stromal Cells from Placenta;  **HSPC:** Hematopoietic CD34^+^ cells; **FIB:** Dermal Fibroblasts.

***Pipeline for the identification of HCC-secreted proteins***

The identification of HCC-secreted proteins was imperative to properly evaluate the interactions between the secreted factors and up-regulated genes in hA-MSCs (Fig. 8). This pipeline can be divided into three phases: 1) identification of up-regulated genes in HCC transcriptome and proteome compared to normal liver, 2) identification of the secreted proteins from the previous phase using experimentally validated dataset, and 3) integration of HCC secretome datasets.

In phase 1, the up-regulated gene sets in HCC compared to the normal liver were obtained from four databases and repositories: GEO datasets, DisGeNET [7], Molecular signatures database (MSigDB) [8], and Dependency Map portal (DepMap) [9]. The cancer dependency map project was built to “catalog and identify biomarkers of genetic vulnerabilities in hundreds of cancer models and tumors” (<https://depmap.org/portal/>). Evidence of GDA in liver carcinoma (UMLS CUI: C2239176) was downloaded from DisGeNET, and only “Biomarker” and “Altered expression” associations were selected to yield 3369 genes out of a total of 3592 genes. From MSigDB, gene sets were downloaded from the curated gene sets collection (CGP: chemical and genetic perturbations). Detailed information about each gene set is presented in (Table S3). Finally, the combined gene dependency scores were downloaded from a collective RNAi screening dataset from multiple sources involving: The Broad Institute Project Achilles, Novartis Project DRIVE, and the Marcotte *et al.* breast cell line dataset [9]. In this dataset, 16 of 24 liver carcinoma cell lines were represented (Dataset S1), and only genes with a D2 gene dependency score > 0 across all liver cancer cell lines were selected. Then, the total scores were log-transformed, followed by z-score normalization, and then ranked based on their z-scores. A total of 3357 genes were identified in 2 or more of the above datasets (Fig.7 A-D) and thus selected for further secretome analysis. All gene comparisons were performed using Entrez ID after gene annotation through the R Bioconductor package “AnnotationDBI” and “Org.Hs.eg.db” and R version 3.5.2 [10].

In phase 2, the aim is to predict which of the previously identified genes are highly expressed on the protein level in HCC serum. Therefore, the Human Protein Atlas (HPA) (<https://www.proteinatlas.org/>) was used [11, 12]. The human secretome dataset from the HPA blood cell atlas was downloaded, specifically proteins characterized as “Secreted in Blood” and “Secreted – unknown location” [13]. An overlapping analysis was performed to identify the proteins that are up-regulated in HCC from the previous phase that overlaps with HPA human secretome. A total of 169 and 20 proteins were identified to be secreted in blood and unknown location respectively. These proteins were thus selected for further analysis.

In phase 3, the aim is to combine the proteins from the previous step with proteins found to be highly expressed in liver cancer according to the pathology atlas in HPA [14, 15], which are also found to be secreted according to the HPA secretome. Only proteins that are “elevated in liver cancer” were selected. A total of 139 out of 457 overexpressed proteins in liver cancer were identified to be secreted in blood. Combining these proteins with the ones identified from the previous step yielded a total of 302 proteins selected for functional enrichment followed by protein-protein interaction (PPI) analysis (Dataset S2).

**Table S3:** Details of gene sets retrieved from MSigDB database used to retrieve the up-regulated gene sets in HCC compared to the normal liver

| **Gene set name** | **No. of genes** | **Exact source** | **Associated dataset** | **Refs.** |
| --- | --- | --- | --- | --- |
| ACEVEDO_LIVER_CANCER_UP | 972 | Table 18S | GSE10842 | [16] |
| BREUHAHN_GROWTH_FACTOR_SIGNALING_IN_ LIVER_CANCER | 23 | Table 1 | N/A | [17] |
| PATIL_LIVER_CANCER | 660 | Suppl. File 4 | N/A | [18] |
| SMITH_LIVER_CANCER | 47 | Fig 1 | N/A | [19] |
| YAMASHITA_LIVER_ CANCER_STEM_CELL_UP | 47 | Table 3S, 4S: Up | GSE5975 | [20] |

**Pipeline for the identification of up-regulated genes in hA-MSCs**

To construct a protein-protein interaction (PPI) network between HCC serum proteins and hA-MSCs up-regulated proteins, a complete profile of up-regulated genes in hA-MSCs should be assembled. Differentially expressed genes sets for hA-MSCs were collected from the following resources: GEO datasets GSE108511 and GSE72332 (Table 1), [21], asc52telo (RRID:CVCL_U602) from [22], and [23]. In Dessels *et al.*, up-regulated genes were selected from passage 3 only in both FBS and pooled Human Platelet Lysate (pHPL) to match the cell culture conditions for hA-MSCs in GEO datasets. Then, up-regulated genes from GEO datasets and Dessels *et al.* were combined to yield a total of 1952 genes. For asc52telo cell line, up-regulated genes having normalized expression (NX) ≥ 1 based on RNA-Seq data obtained from HPA (<https://www.proteinatlas.org/humanproteome/cell/cell+line>). Enriched, enhanced, group enriched genes were selected yielding a total of 505 genes. For single-cell RNA-Seq data, the scaled expression matrix after batch and cell cycle effect removal was downloaded from supplementary data [1]. The expression matrix accounts for normalized expression in 24,358 cultured human hA-MSCs.

For each gene, the normalized expression values across all the cells were added, and only genes having a positive sum were selected. Then, the total sum was scaled and then ranked based on the z-score. Genes having z-score < 0 were excluded. Finally, an overlapping analysis was conducted on the mentioned gene sets, and a total of 533 genes (Dataset S3) were identified in 2 or more of the above datasets (Fig.7 E). These genes were selected for functional enrichment followed by PPI analysis. All of the aforementioned analyses were performed using R version 3.5.2. All gene comparisons were performed using Entrez ID after gene annotation through the R Bioconductor package “AnnotationDBI” and “Org.Hs.eg.db” and R version 3.5.2 [10] .

**Supplementary Figures**


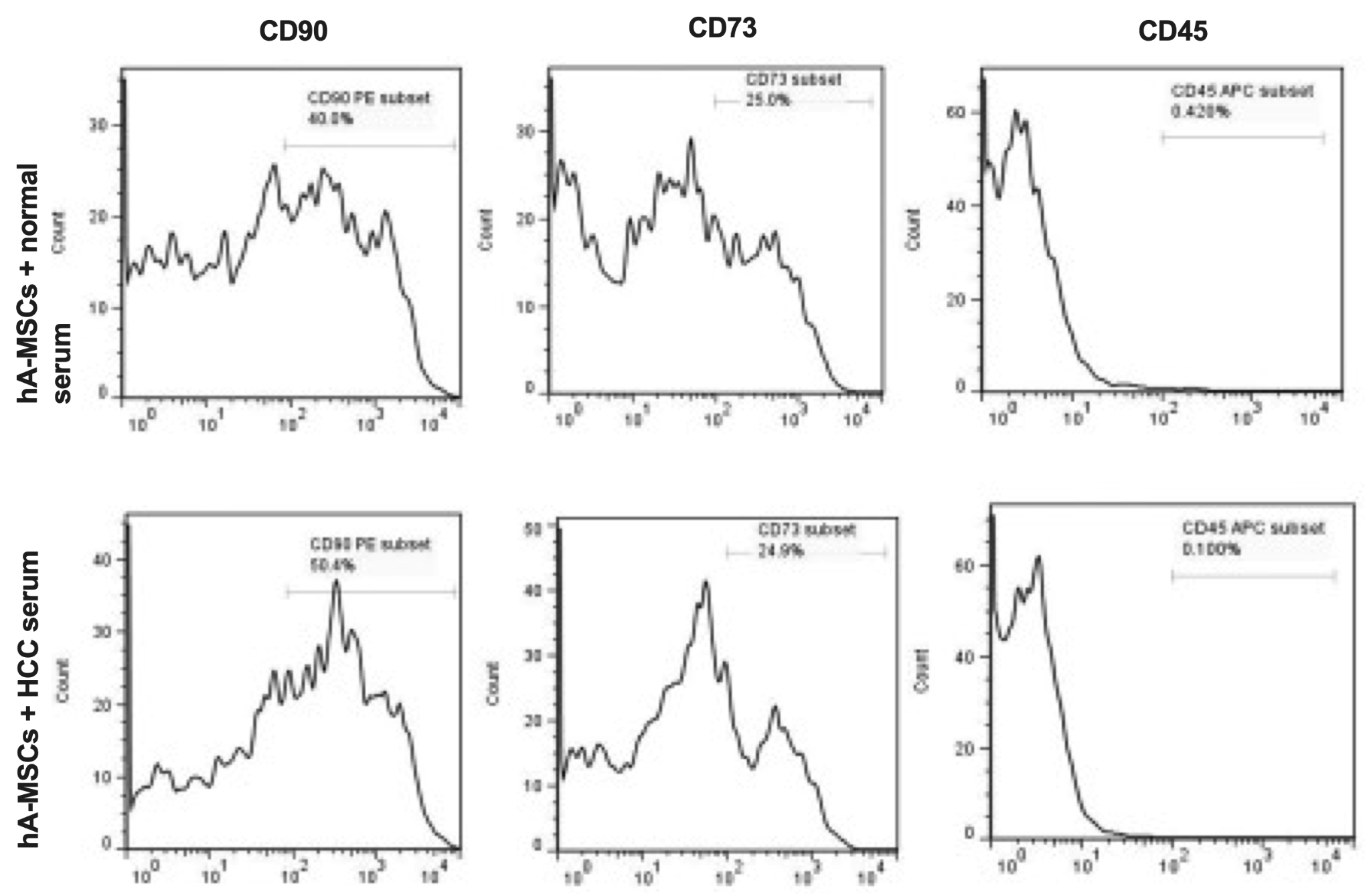
**Fig.S1: Effect of serum from HCC patients on the phenotypic characteristics of hA-MSCs.** (A-B) Flow cytometry analysis for CD90, CD73, and CD45 surface markers in normal and HCC serum-treated hA-MSCs respectively. No significant difference was observed.


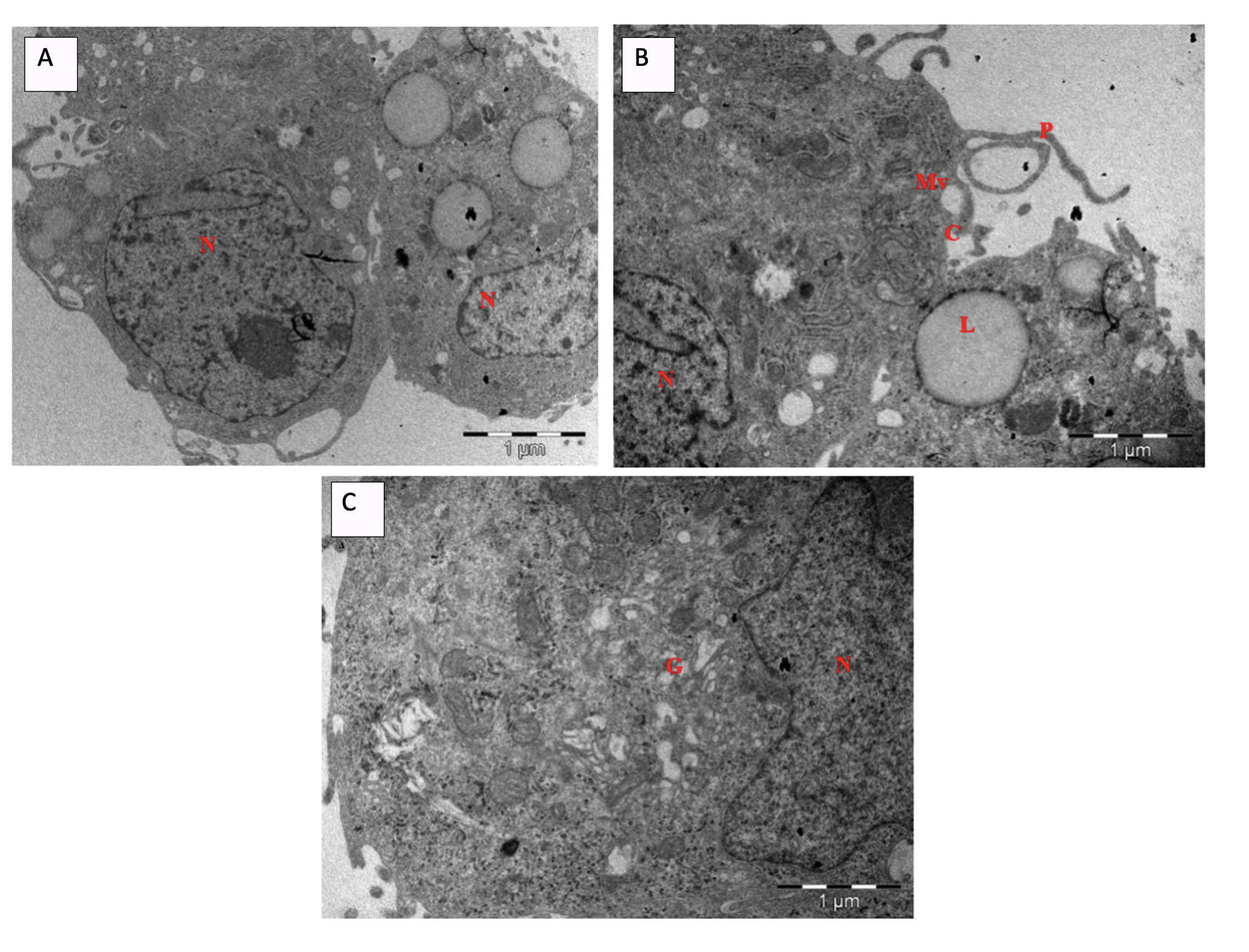


**Fig.S2: An electron micrograph of Huh-7 cells supplemented with serum from HCC patients after 6 days of treatment**. **(A)** Newly dividing cells with visible cytokinesis, irregularly shaped nucleus (N), and 2 nucleoli (Nu) in each nucleus. **(B)** Part of two cells showing cytokinesis (C), lipid droplet (L), elongated process (P), irregularly shaped nucleus (N), and microvilli (Mv). **(C)** A magnified part of (B) showing multiple active Golgi apparatus.


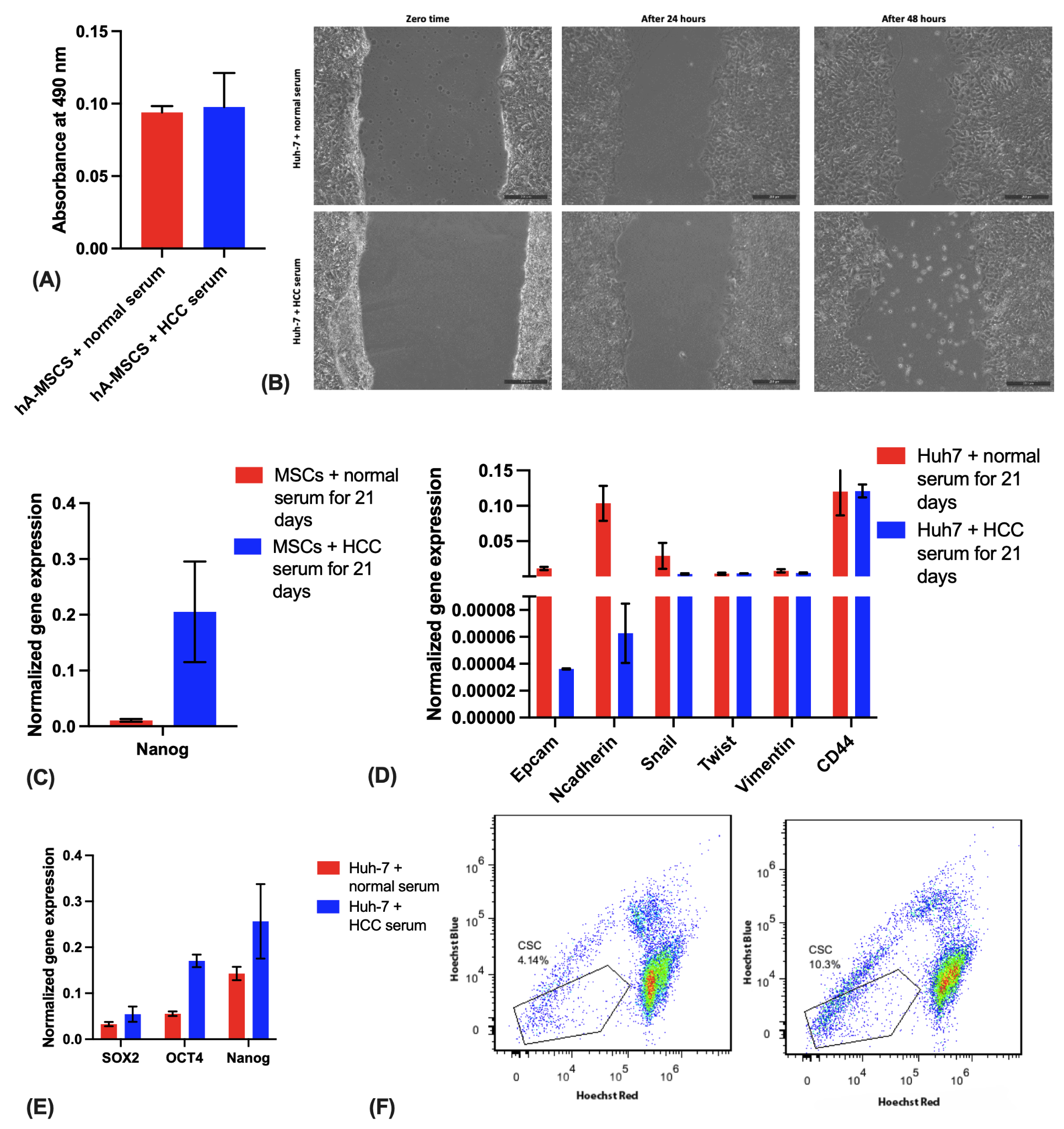


**Fig. S3:** Assessment of hA-MSCs and Huh-7 cells treated with either normal or HCC serum for 21 days. **(A)** MTT proliferation assay for hA-MSCs after 21 days of treatment. During 48 hours wound closure assay, pictures were taken using inverted fluorescence microscope. Scale bars(200 µm) were added for: **(A)** hA-MSCs treated with serum from normal volunteers at zero time, 24 hours, and 48 hours. **(B)** hA-MSCs treated with serum from HCC patients at zero time, 24 hours, and 48 hours.**(B)** Scratch assay for hA-MSCs treated with normal and HCC serum to assess their migration potential.**(C,D,E)** Normalized expression in normal and HCC serum-treated hA-MSCs for 21 days **(C)**, Huh-7 cells **(D,E)** .


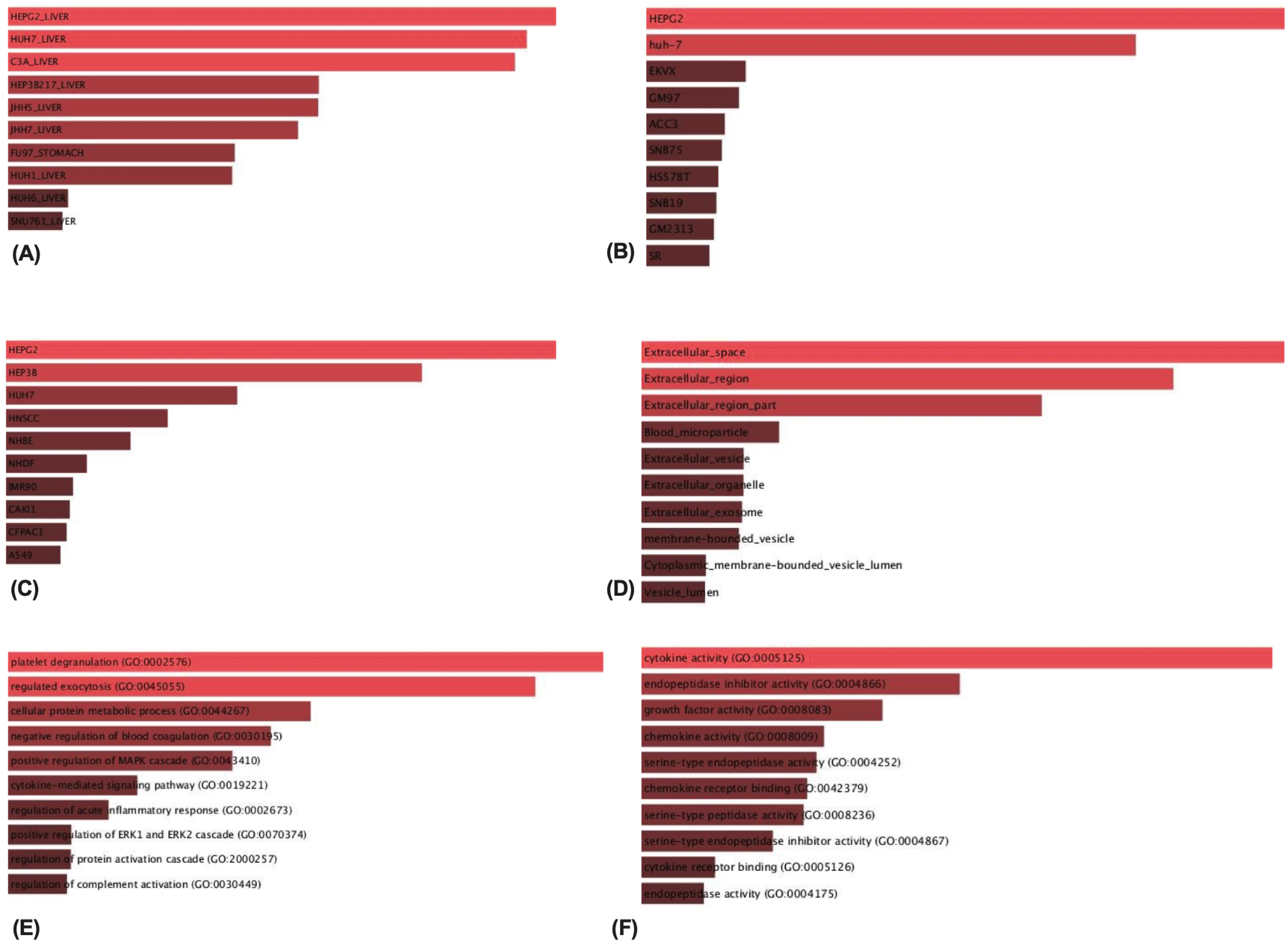


**Fig. S4:** Cancer Cell Line Encyclopedia Enrichment analysis. **(A)** Bar graph displaying the top 10 enriched CCLE cell lines for the HCC secretome gene set comprised of 302 genes. **(B)** NCI-60 Enrichment analysis. Bar graph displaying the top 10 enriched NCI-60 cell lines for the HCC secretome gene set comprised of 302 genes. **(C)** ARCHS4 Cell lines Enrichment analysis. Bar graph displaying the top 10 enriched ARCHS4 cell lines for the HCC secretome gene set comprised of 302 genes. **(D)** Compartments enrichment analysis. Bar graph displaying the top 10 enriched compartments based in the Jensen database for the HCC secretome gene set comprised of 302 genes. **(E)** GO Biological Process enrichment analysis. Bar graph displaying the top 10 enriched GO BP terms for the HCC secretome gene set comprised of 302 genes. **(F)** GO Molecular Function enrichment analysis. Bar graph displaying the top 10 enriched GO MF terms for the HCC secretome gene set comprised of 302 genes. The bars are ranked according to adjusted p-value based on the fisher-exact test.

1. Chen, E.Y., et al., *Enrichr: interactive and collaborative HTML5 gene list enrichment analysis tool.* BMC bioinformatics, 2013. **14**(1): p. 1-14.

2. Gundersen, G.W., et al., *GEO2Enrichr: browser extension and server app to extract gene sets from GEO and analyze them for biological functions.* Bioinformatics, 2015. **31**(18): p. 3060-3062.

3. Jiang, Y., et al., *Peptidase inhibitor 15 as a novel blood diagnostic marker for cholangiocarcinoma.* EBioMedicine, 2019. **40**: p. 422-431.

4. Schulze, K., et al., *Exome sequencing of hepatocellular carcinomas identifies new mutational signatures and potential therapeutic targets.* Nature genetics, 2015. **47**(5): p. 505-511.

5. Alhattab, D., et al., *An insight into the whole transcriptome profile of four tissue-specific human mesenchymal stem cells.* Regenerative medicine, 2019. **14**(9): p. 841-865.

6. Rosón, B., et al., *Insights into the human mesenchymal stromal/stem cell identity through integrative transcriptomic profiling.* 2016.

7. Sanz, F., et al., *DisGeNET: a comprehensive platform integrating information on human disease-associated genes and variants.* Nucleic Acids Research, 2017, vol. 45, núm. D1, p. 833-839, 2017.

8. Liberzon, A., et al., *Molecular signatures database (MSigDB) 3.0.* Bioinformatics, 2011. **27**(12): p. 1739-1740.

9. McFarland, J.M., et al., *Improved estimation of cancer dependencies from large-scale RNAi screens using model-based normalization and data integration.* Nature communications, 2018. **9**(1): p. 4610.

10. Pages, H., et al., *Package ‘AnnotationDbi’.* Bioconductor Packag. Maint, 2017.

11. Uhlén, M., et al., *Tissue-based map of the human proteome.* Science, 2015. **347**(6220): p. 1260419.

12. Uhlén, M., et al., *A human protein atlas for normal and cancer tissues based on antibody proteomics.* Molecular & cellular proteomics, 2005. **4**(12): p. 1920-1932.

13. Uhlén, M., et al., *The human secretome.* Science signaling, 2019. **12**(609): p. eaaz0274.

14. Uhlen, M., et al., *A pathology atlas of the human cancer transcriptome.* Science, 2017. **357**(6352): p. eaan2507.

15. Kampf, C., et al., *The human liver‐specific proteome defined by transcriptomics and antibody‐based profiling.* The FASEB Journal, 2014. **28**(7): p. 2901-2914.

16. Acevedo, L.G., et al., *Analysis of the mechanisms mediating tumor-specific changes in gene expression in human liver tumors.* Cancer Research, 2008. **68**(8): p. 2641-2651.

17. Breuhahn, K., T. Longerich, and P. Schirmacher, *Dysregulation of growth factor signaling in human hepatocellular carcinoma.* Oncogene, 2006. **25**(27): p. 3787-3800.

18. Patil, M.A., et al., *An integrated data analysis approach to characterize genes highly expressed in hepatocellular carcinoma.* Oncogene, 2005. **24**(23): p. 3737-3747.

19. Smith, M.W., et al., *Identification of novel tumor markers in hepatitis C virus-associated hepatocellular carcinoma.* Cancer research, 2003. **63**(4): p. 859-864.

20. Yamashita, T., et al., *EpCAM-positive hepatocellular carcinoma cells are tumor-initiating cells with stem/progenitor cell features.* Gastroenterology, 2009. **136**(3): p. 1012-1024. e4.

21. Dessels, C., M.A. Ambele, and M.S. Pepper, *The effect of medium supplementation and serial passaging on the transcriptome of human adipose-derived stromal cells expanded in vitro.* Stem Cell Research & Therapy, 2019. **10**(1): p. 1-17.

22. Wolbank, S., et al., *Telomerase immortalized human amnion-and adipose-derived mesenchymal stem cells: maintenance of differentiation and immunomodulatory characteristics.* Tissue Engineering Part A, 2009. **15**(7): p. 1843-1854.

23. Liu, X., et al., *Single-cell RNA-seq of cultured human adipose-derived mesenchymal stem cells.* Scientific data, 2019. **6**(1): p. 1-8.
